# GLP-1/GIP/GCG receptor triagonist (IUB447) enhances insulin secretion via GLP-1 receptor and Gαq signalling pathway in mice

**DOI:** 10.1007/s00125-025-06525-0

**Published:** 2025-09-09

**Authors:** Pascale C. F. Schreier, Philipp Beyerle, Severin Boulassel, Andreas Beck, Aaron Novikoff, Peter S. Reinach, Ingrid Boekhoff, Andreas Breit, Arthur Neuberger, Timo D. Müller, Alberto Cebrian Serrano, Thomas Gudermann, Noushafarin Khajavi

**Affiliations:** 1 Walther Straub Institute of Pharmacology and Toxicology, LMU Munich, Munich, Germany; 2 Institute of Experimental and Clinical Pharmacology and Toxicology, Saarland University, Homburg, Germany; 3 Institute of Diabetes and Obesity, Helmholtz Munich, Munich, Germany; 4 German Center for Diabetes Research (DZD), Neuherberg, Germany; 5 Wenzhou Medical University, Ophthalmology Department, Wenzhou, P. R. China; 6 German Center for Lung Research (DZL), Munich, Germany

**Keywords:** GLP-1 receptor, Gαq signalling, Insulin secretion, Pancreatic islet, Triagonist, TRPM5

## Abstract

**Aims/hypothesis:**

Unimolecular peptides targeting the receptors for glucagon-like peptide-1 (GLP-1), glucose-dependent insulinotropic polypeptide (GIP) and glucagon (GCG) have been shown to improve glycaemic management in both mice and humans. Yet the identity of the downstream signalling events mediated by these peptides remain to be elucidated. Here, we aimed to assess the mechanisms by which a validated peptide triagonist for GLP-1/GIP/GCG receptors (IUB447) stimulates insulin secretion in murine pancreatic islets.

**Methods:**

Islets were isolated from wild-type (WT), *Gipr*-knockout (*Gipr*^−/−^), *Gcgr*-knockout (*Gcgr*^−/−^), *Glp-1r* (also known as *Glp1r*)/*Gipr* double-knockout and *Trpm5*-knockout (*Trpm5*^−/−^) mice, followed by assessment of beta cell function and insulin secretion in response to mono- and multi-agonist administration. Metabolic phenotypes of WT and *Trpm5*^−/−^ mice under chow and high-fat diets were investigated following triagonist application.

**Results:**

The triagonist promoted glucose-stimulated insulin secretion (GSIS) to a greater degree than co-administration of conventional mono-agonists in WT mouse islets. The triagonist-induced increase in GSIS was unchanged in the absence of either *Gipr* or *Gcgr*. However, the triagonist failed to enhance insulin secretion in islets lacking both *Glp-1r* and *Gipr* and upon treatment with the GLP-1 receptor-specific antagonist exendin-3 (9–39). Similarly, the specific blocking of Gαq signalling with YM254890 or transient receptor potential melastatin 5 (TRPM5) with triphenylphosphine oxide (TPPO) suppressed the triagonist-induced enhancement of GSIS. In vivo assessment of high-fat-fed *Trpm5*^−/−^ mice demonstrated the absence of triagonist-induced therapeutic effects on glycaemic management.

**Conclusions/interpretation:**

Triagonist-induced augmentation of GSIS is primarily mediated through its interaction with the GLP-1 receptor and subsequent activation of the Gαq–TRPM5 signalling pathway. Given that Gαq is a key player in the amplification of GSIS, particularly under diabetic conditions, these findings highlight a GLP-1 receptor-centric pharmacological profile that underlies the potent effects of this multi-receptor agonist.

**Graphical Abstract:**

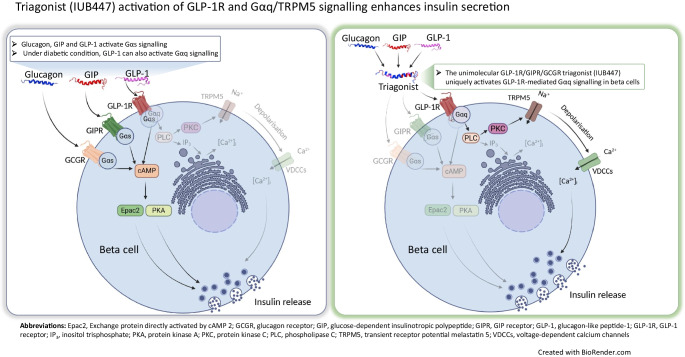

**Supplementary Information:**

The online version of this article (10.1007/s00125-025-06525-0) contains peer-reviewed but unedited supplementary material.


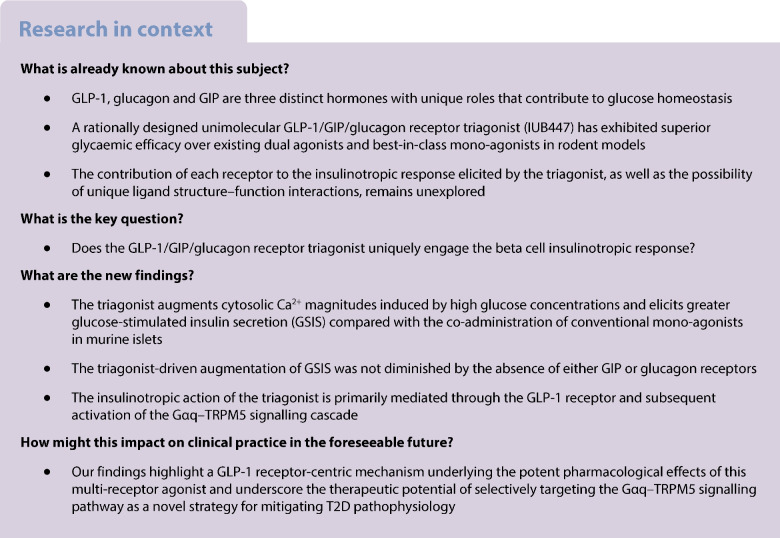


## Introduction

Combinational pharmacotherapies provide a promising approach for the treatment of obesity and type 2 diabetes. Co-agonism, or tri-agonism, of separate therapeutic targets leverage strategic combinatorial synergism to amplify satiety, body-weight lowering and glucoregulatory benefits. Previous studies demonstrate that sequence hybridisation of glucagon-like peptide-1 (GLP-1), glucose-dependent insulinotropic polypeptide (GIP) and glucagon (GCG) into a unimolecular triple agonist (GLP-1/GIP/GCG) effectively enhances glycaemic management relative to conventional mono-agonist treatments in diet-induced obese mice [1, 2]. These desirable effects are prompting efforts to clarify the identity of signalling pathway mechanisms that mediate such responses in health and disease.

Long-acting GLP-1 receptor (GLP-1R) mono-agonists have been developed to engage in beta cell glucose-dependent insulinotropic effect, while also acting within key feeding regions of the central nervous system to influence satiety. GCG is primarily known for its essential role in maintaining glucose homeostasis through its activation of hepatic glycogenolysis and gluconeogenesis during periods of starvation [3]. However, long-acting GCG receptor (GCGR) agonists have also been shown to dose-dependently stimulate insulin secretion and enhance energy expenditure and fatty acid oxidation, leading to substantial weight loss in rodents [4, 5]. These findings led to the strategic development of a unimolecular GLP-1/GCG co-agonist that exploits the energy expenditure and lipolytic properties of GCG and the appetite-suppressing and insulinotropic properties of GLP-1 [6]. A cautionary note on GLP-1-based pharmacology is the limitation on dose escalation due to corresponding increases in gastrointestinal tolerability issues. To mitigate this drawback, the pharmacological administration of GIP, primarily known for its insulinotropic and mild appetite-suppressing properties [7], is also suggested to have an anti-emetic effect, potentially allowing GIP co-agonism to increase tolerability to the GLP-1 component [8].

The complementary nature of the desirable attributes in GLP-1, GIP and GCG led to the creation of a unimolecular hybridised triagonist with retained and balanced activity across the GLP-1R, GIP receptor (GIPR) and GCGR [1]. Despite definitive evidence suggesting that the triagonist offers a promising therapeutic option for the treatment of type 2 diabetes, the specific cellular mechanism by which the enhanced efficacy in glycaemic management is attained remains unclear.

The GLP-1R/GIPR/GCGR triagonist IUB447 enhances glycaemic management through concurrent activation of GLP-1R, GIPR and GCGR, and corresponds to the triagonist previously reported by Finan et al [1]. Recently, we found that triagonist-induced enhancement of insulin secretion exceeds levels observed with the loose co-administration of the three individual agonists in murine islets [9]. This difference raises the first question: does the superior triagonist effect stem from an additional mode of action beyond the activation of the three receptors?

Accumulating evidence suggests that GLP-1R activates both Gαq and Gαs signalling pathways, whereas GIPR and GCGR primarily couple to Gαs [10–12]. Under diabetic conditions, persistent beta cell depolarisation shifts GLP-1R signalling toward Gαq dominance, preserving its insulinotropic effect [13]. This shift may account for why GLP-1R agonists remain a useful glucose-lowering therapy, whereas GIPR loses insulinotropic efficacy [13–15]. Given the potential loss of effectiveness in Gαs signalling in diabetic conditions and that both GIP and GCG seem to primarily act through Gαs signalling, it raises a second question: is the effect of triagonist on glucose-stimulated insulin secretion (GSIS) mediated solely via GLP-1R?

In this study, we sought to unravel the mechanisms through which the triagonist enhances insulin secretion in murine islets.

## Methods

The sources of reagents are listed in electronic supplementary material (ESM) Table 1.

### Mouse strains

*Gipr*^−/−^, *Gcgr*^−/−^ and *Glp-1r* (also known as *Glp1r*)*/Gipr* double-knockout (KO) mice were generated. sgRNAs against exons 4 and 5 of the *Gipr*, *Glp-1r* and *Gcgr* genes (ESM Table 2) were designed using CRISPOR tool (www.crispor.tefor.net), in vitro transcribed (E3322, NEB) and purified using the MEGAclear Kit. C57BL/6n female mice at 4 weeks of age were super ovulated and mated with C57BL/6n studs. Fertilised oocytes were electroporated (NEPA21) with a total concentration of 200 ng/μl sgRNAs and 200 ng/μl recombinant NLS-Cas9 protein. Electroporated zygotes were surgically implanted into recipient CD1 females. For genotyping, ear biopsies were lysed and genomic DNA was purified. Target genes were amplified using primers listed in ESM Table 3 and verified by Sanger sequencing to confirm CRISPR-induced deletions. Founders carrying the following deletion alleles were bred to generate single and double KO lines: a five-nucleotide (nt) deletion in exon 5 of *Gipr*; a 62-nt deletion in exon 4 of *Gcgr*; and a 495-nt deletion spanning exon 4, intron 4 and exon 5 of *Glp-1r*. CRISPR-Cas9-induced deletions result in a premature stop codon at the exon 5 (*Gipr* and *Gcgr*) and 6 (*Glp-1r*). To genotype the *Gipr* deletion, specific primers binding on the wild-type (WT) or deleted DNA region were designed and established (*Gipr*-9-nt forward and reverse, and *Gipr* WT reverse primers) (ESM Table 3). *Gcgr* and *Glp-1r* deletions were genotyped by PCR and resolved on agarose gel electrophoresis until double bands were obtained.

Transient receptor potential melastatin 5 (TRPM5) KO (B6;129-*Trpm*5tm1Csz/J) mice were obtained from Jackson Laboratory (https://www.jax.org/strain/013068). Heterozygous *Trpm5*^+/−^ mice were bred to obtain age- and sex-matched homozygous wild-type (WT) and homozygous *Trpm5*^−/−^ mice. Genotyping was performed using the One Step Mouse Genotyping Kit. *Trpm5* transgene inheritance was confirmed by PCR (primers in ESM Table 3; protocol in ESM Table 4).

Mice were fed a normal chow diet (LFD) or a high-fat diet (HFD) (D12451 or D12331), with 45% and 58% energy from fat, respectively. After being fed the HFD for 16 weeks, mice were treated every other day with either vehicle or triagonist (3 nmol/kg) for 3 weeks. Mice were single- or group-housed under a 12 h light–dark cycle at 22°C with ad libitum access to food and water. Mice were randomly assigned to experimental groups using a random number generator. This ensured unbiased allocation and reduced potential confounding factors. Experimenters were masked to group assignments during outcome assessment to minimise bias. No animals, samples, or data points were excluded from the analysis. All experiments complied with the EU Animal Welfare Act and were approved by the District Government of Upper Bavaria, Germany (permit no. 55.2–2532. Vet_02-21-75).

### Characterisation of glucose homeostasis

GTTs were performed as previously described [16]. Mice were fasted for 16 h and injected intraperitoneally with glucose (2 g/kg body weight). Blood glucose was measured using a glucometer (TheraSense FreeStyle) at 0, 15, 30, 60 and 120 min. Plasma was collected post-euthanasia, stored at −80°C, and analysed for insulin and GCG levels by ELISA.

### Islet isolation and determination of insulin secretion

Islet isolation was performed as previously described [9]. In brief, the pancreas was perfused via the common bile duct with collagenase-P (0.3 mg/ml). Isolated islets were cultured for 48 h in RPMI 1640 before use in functional assays. For GSIS, islets were equilibrated for 1 h in KRB buffer (ESM Table 5) with 2.8 mmol/l glucose. Next, islets were incubated for 1 h in 20 mmol/l glucose supplemented with agonists in the presence or absence of blockers. Exendin-3 (GLP-1 blocker), LY2409021 (GCG blocker), YM-254890 (Gαq blocker), MDL-12330A (adenylate cyclase blocker), calphostin C (protein kinase C [PKC] blocker) and TPPO (TRPM5 blocker) were used as specific inhibitors. Insulin was quantified in the supernatant fraction using ELISA.

### Cell culture

MIN6 cells were provided by P.-O. Berggren and B. Leibiger, Karolinska Institutet, Stockholm, Sweden. Cells were cultured at 37°C with 5% CO_2_ in DMEM supplemented with 10% FBS, 100 U/ml penicillin, 100 μg/ml streptomycin and 75 μmol/l β-mercaptoethanol. The cells were routinely tested and no mycoplasma contamination was detected. Authentication of the MIN6 cell line was performed based on morphology and functional characteristics.

### Calcium imaging

Changes in intracellular Ca^2+^ concentration ([Ca^2+^]_i_) were recorded as previously described [17]. Confocal imaging was performed using a Zeiss LSM 510 Meta system with a 63×/NA1.2 water immersion objective. Regions of interest were selected using LSM software, and calcium dynamics were monitored via Fluo-4 fluorescence (excitation λ: 488 nm; emission λ: 500–550 nm). Images (8-bit, 512 × 512 pixels) were acquired every 5 s.

### α Screen-based detection of intracellular cAMP

Islets were plated in clear-bottom 96-well plates with KRB containing 2.8 mmol/l glucose and stimulated for 20 min at 37°C with KRB (20 mmol/l glucose) plus IBMX (500 µmol/l), either alone or combined with forskolin (10 µmol/l), GLP-1 (1 nmol/l) or triagonist (1 nmol/l). Stimulation was terminated by adding lysis buffer containing acceptor beads and biotinylated cAMP. After 90 min, donor beads were added and incubated for 60 min. Acceptor bead emission (λ 570 ± 100 nm) was measured after donor bead excitation (λ 680 ± 40 nm) using a ClarioStar plate reader (BMG, Offenburg, Germany).

### Morphological analysis

Islet morphology was assessed by H&E staining of 10 μm cryosections and whole-islet immunofluorescence. Antibodies are shown in ESM Table 6; antibody validation was carried out according to the manufacturers’ specifications, and all relevant information, including recommended dilutions and buffer compositions, was provided by the manufacturers. Imaging was done using a MetaSystems scanner with a Zeiss Imager Z.2 microscope, and quantitative analysis with FIJI 2.16.0 [18].

### Statistics

Data were expressed as mean ± SEM. A *p* value less than 0.05 was considered significant. Graphical presentations and statistics were obtained by Prism software (version 9.0.1; GraphPad). For comparison of two groups, *p* values were calculated by the unpaired two-tailed Student’s *t* test for parametric distribution or Mann–Whitney test for non-parametric distribution. For three or more groups, one-way ANOVA with Bonferroni multiple comparison were used for parametrically distributed data. Glucose tolerance tests were compared using two-way ANOVA with Bonferroni multiple comparison.

## Results

### Triagonist effectively reverses glucose metabolism dysfunction induced by HFD in WT mice

**Fig. 1:**
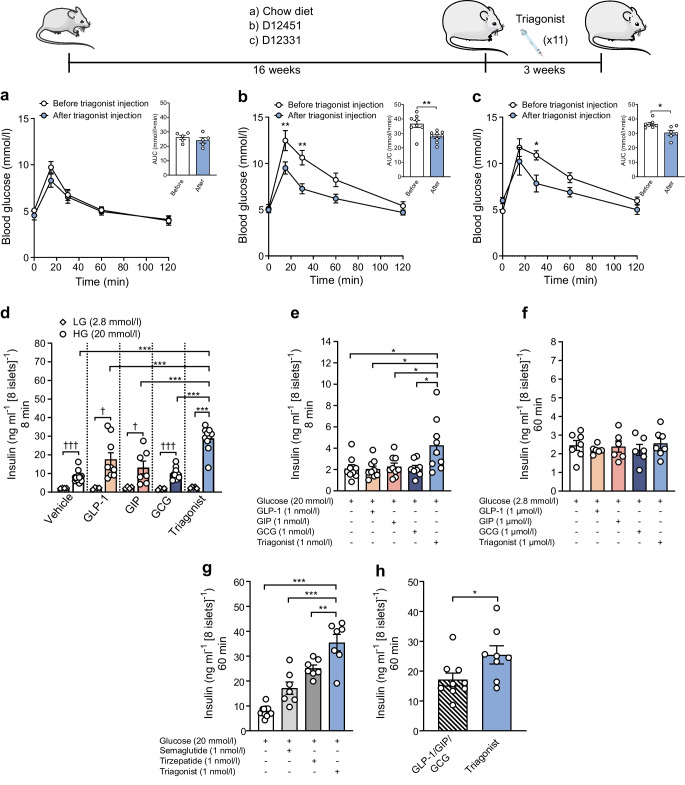
Triagonist improves glucose metabolism and GSIS in mice. (**a**–**c**) Ten- to twelve-week-old WT C57BL/6 mice were divided into three groups and assigned to different diets. A GTT was performed before and after 3 weeks of treatment with 3 nmol/kg triagonist given by i.p. injection every other day. (**a**–**c**) One group was maintained on chow diet (LFD) (*n*=7) (**a**), while the other two groups were placed on different HFDs, either D12451 (**b**) or D12331 (**c**), for 16 weeks (*n*≥7 per group). For the GTT, mice were fasted overnight. Blood glucose levels (mmol/l) before and within 2 h after i.p. injection of glucose (2 g/kg body weight) are shown, together with the AUC for glucose (in mmol/l × min). (**d**, **e**) Insulin secretion (ng ml^−1^ [8 islets]^−1^) was assessed in isolated islets from WT mice on LFD (*n*≥3 mice, measured in duplicate). After 1 h pre-incubation in KRB with 2.8 mmol/l glucose, islets were stimulated with 2.8 mmol/l glucose (LG) or 20 mmol/l glucose (HG) supplemented with mono- or multi-agonist (1 nmol/l each). Insulin content was determined, by ELISA, in supernatant fractions collected at 60 min (**d**) or 8 min (**e**) after stimulation. (**f**) Insulin secretion was assessed following 1 h stimulation with a glucose concentration of 2.8 mmol/l, supplemented with mono- or multi-agonist (1 µmol/l each). (**g**) Insulin secretion was assessed following 1 h stimulation with glucose 20 mmol/l, supplemented with the glucose-lowering drugs semaglutide (1 nmol/l) or tirzepatide (1 nmol/l), in comparison with the response elicited by the triagonist (1 nmol/l). (**h**) Insulin secretion was assessed following 1 h stimulation with 20 mmol/l glucose, supplemented with either a triagonist or co-administration of mono-agonists (1 nmol/l each). Data show means ± SEM, and statistical differences were assessed by two-way ANOVA (**a**–**c**, blood glucose), unpaired two-tailed Student’s *t* test (**a**–**c**, AUC for glucose; **h**) or one-way ANOVA (**d**–**g**). Circles in bar graphs represent single values. *p<0.05; **p<0.01; ***p<0.001 for indicated comparisons; ^†^*p*<0.05, ^†††^*p*<0.001 for HG vs LG

Ten- to twelve-week-old WT (C57BL/6) mice were fed LFD or obesogenic diets (D12451 or D12331) for 16 weeks. Triagonist-induced improvements in glucose tolerance were assessed relative to pre-treatment levels within each diet group. Both HFDs markedly impaired glucose tolerance (ESM Fig. 1a, b). Three weeks of the triagonist treatment improved glucose tolerance in HFD-fed mice, with minimal effect in LFD-fed controls (Fig. 1a–c).

### Triagonist increases GSIS in primary islets

To assess the effects of the triagonist and mono-agonists on GSIS, single islets from WT mice fed an LFD were exposed to a switch from low glucose (2.8 mmol/l) to high glucose (20 mmol/l), with or without agonists. Supernatant fractions were collected at 8 and 60 min. The glucose switch alone induced a ~fourfold increase in insulin secretion after 60 min; GLP-1, GIP and GCG (each at 1 nmol/l) enhanced GSIS by ~eightfold, ~fivefold and ~fourfold, respectively, whereas the triagonist (1 nmol/l) elicited an ~11-fold increase (Fig. 1d). Notably, after 8 min, only the triagonist significantly increased GSIS (~twofold), with the mono-agonists having no effect (Fig. 1e). Elevating the concentration of triagonist to 1 µmol/l had no effect on insulin secretion at a glucose concentration of 2.8 mmol/l (Fig. 1f), ruling out potential islet-derived hypoglycaemia and reinforces glucose-dependency as a mode of action. The triagonist similarly enhanced GSIS in MIN6 cells, excluding alpha cell-derived GCG as underlying mechanism of its superior effect (ESM Fig. 2a). The stimulatory effect of triagonist on GSIS surpassed that of the two glucose-lowering drugs, semaglutide and tirzepatide, in WT mouse islets (Fig. 1g). Triagonist-enhanced GSIS was up to 50% greater than that seen with semaglutide and 25% greater than that seen with tirzepatide. Surprisingly, the triagonist increased GSIS by up to 35% compared with loose co-administration of GLP-1, GIP and GCG, in both mouse islets and MIN6 cells (Fig. 1h and ESM Fig. 2b).

### Triagonist enhances cytosolic Ca^2+^ responses induced by high glucose

**Fig. 2:**
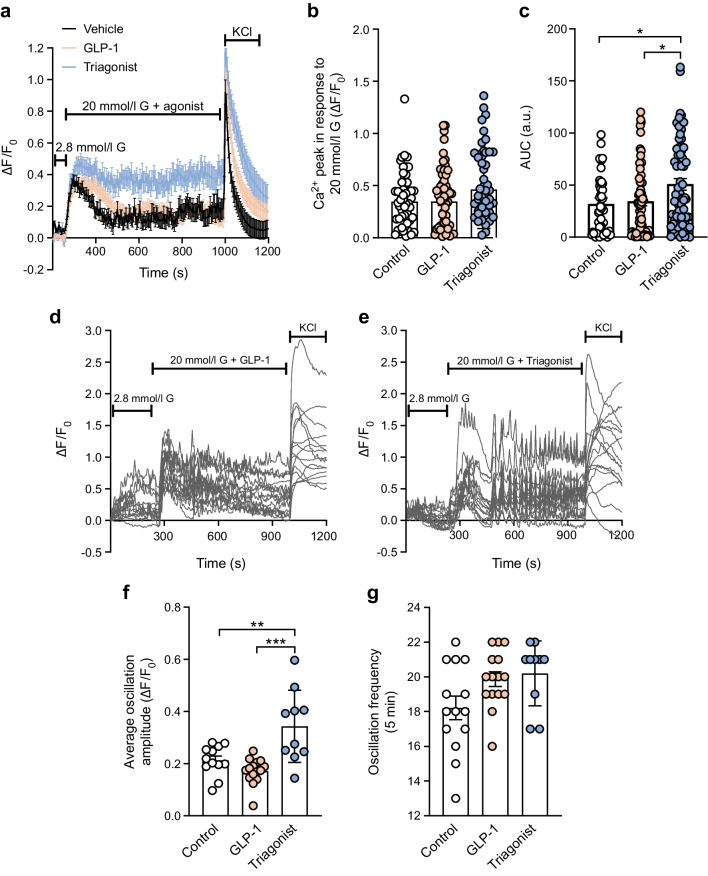
Triagonist increases cytoplasmic Ca^2+^ concentration in beta cells. (**a**) Intact WT islets (*n*≥50 islets per condition, isolated from at least ten mice) were loaded with 3 µmol/l Fluo-4-AM and alterations in [Ca^2+^]_i_ of individual cells were monitored by confocal microscopy after increasing the extracellular glucose concentration from 2.8 to 20 mmol/l in the presence of GLP-1 (1 nmol/l) or triagonist (1 nmol/l). KCl (30 mmol/l) was used as a positive control. F_0_ was calculated as the mean fluorescence intensity during the final 2 min prior to stimulation with glucose 20 mmol/l + agonist to minimise potential bias from differences in dye loading, photobleaching or probe stabilisation. (**b**, **c**) Average of Ca^2+^ influx peaks assessed from baseline after glucose stimulation (**b**) and AUC (only during agonist application, **c**). (**d**, **e**) Individual traces of Fluo-4 intensity of single islet cells in the presence of GLP-1 (1 nmol/l, **d**) or triagonist (1 nmol/l, **e**), elicited by excitation at λ 480 nm (indicative of [Ca^2+^]_i_). (**f**) Average [Ca^2+^]_i_ oscillation amplitude calculated based on alteration in Fluo-4 intensity in single cells following an initial peak after 20 mmol/l glucose stimulation or in the presence of different agonists (*n*≥10 cells). (**g**) Representative changes in frequency of [Ca^2+^]_i_ oscillations in 5 min following an initial peak after 20 mmol/l glucose stimulation or in the presence of different agonists (*n*≥10 cells). The data are presented as means ± SEM (circles in bar graphs represent single values) and statistical differences were assessed by one-way ANOVA (**b**, **c**, **f**, **g**). *p<0.05, **p<0.01, ***p<0.001. a.u., arbitrary units; G, glucose

We next examined whether triagonist-enhanced GSIS is associated with increased Ca^2+^ mobilisation in beta cells. At 2.8 mmol/l glucose, beta cells exhibited low and relatively stable [Ca^2+^]_i_ levels, whereas 20 mmol/l glucose triggered transient Ca^2+^ increases, combined with frequent oscillatory patterns (Fig. 2a). The initial Ca^2+^ peak following glucose stimulation was comparable across control (vehicle), GLP-1 (1 nmol/l) and triagonist (1 nmol/l) (Fig. 2b). After cessation of Ca^2+^ transient activity, [Ca^2+^] remained elevated with the triagonist compared with control and GLP-1 (Fig. 2c). Notably, the amplitude of oscillations during the plateau phase was significantly higher with triagonist treatment (Fig. 2d–f), whereas oscillation frequency was only slightly increased with GLP-1 and triagonist relative to control (Fig. 2g).

### Triagonist enhances GSIS mainly through GLP-1R activation

**Fig. 3:**
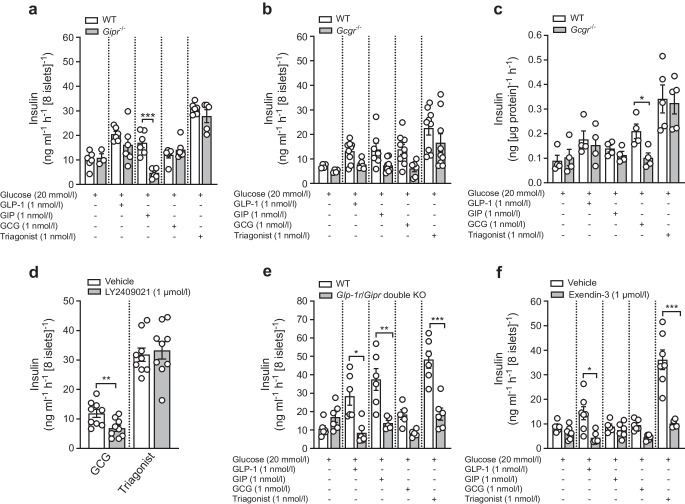
Triagonist primarily enhances GSIS through activation of the GLP-1R. Insulin secretion was assessed after 1 h pre-incubation in KRB with 2.8 mmol/l glucose; islets were stimulated with 20 mmol/l glucose, supplemented with mono- or multi-agonist (1 nmol/l each). (**a**, **b**) Insulin secretion (ng ml^−1^ h^−1^ [8 islets]^−1^) in isolated islets from WT mice vs either *Gipr*^−/−^ mice (**a**) or *Gcgr*^−/−^ mice (**b**) on a chow diet (LFD) (*n*≥3 mice per condition). (**c**) Insulin secretion normalised to protein content (ng [µg protein]^−1^ h^−1^) in isolated islets from WT and *Gcgr*^−/−^ mice on LFD (*n*≥4 mice per condition). (**d**) Insulin secretion (ng ml^−1^ h^−1^ [8 islets]^−1^) in isolated islets from WT mice on LFD (*n*≥5 mice per condition) after 1 h pre-incubation in KRB with 2.8 mmol/l glucose in the presence or absence of 1 µmol/l LY2409021. Islets were stimulated with 20 mmol/l glucose ± blocker, supplemented with GCG or triagonist (1 nmol/l each). (**e**) Insulin secretion (ng ml^−1^ h^−1^ [8 islets]^−1^) in isolated islets from WT, *Glp-1r/Gipr* double KO mice on LFD (*n*≥3 mice per condition). (**f**) Insulin secretion (ng ml^−1^ h^−1^ [8 islets]^−1^) in isolated islets from WT mice on LFD (*n*≥5 mice per condition) after 1 h pre-incubation in KRB with 2.8 mmol/l glucose in the presence or absence of 1 µmol/l exendin-3. Islets were stimulated with 20 mmol/l glucose ± blocker, supplemented with mono- or multi-agonist (1 nmol/l each). Insulin content in supernatant fractions were collected 60 min after stimulation and ELISA was used to determine the insulin content in the fraction. The data are presented as means ± SEM (circles in bar graphs represent single values) and statistical differences were assessed by unpaired two-tailed Student’s *t* test. *p<0.05, **p<0.01, ***p<0.001

Next, we assessed the relative contribution made by GLP-1R, GIPR and GCGR towards mediating triagonist-induced responses. Accordingly, triagonist-induced GSIS was evaluated in islets from *Gipr*^−/−^, *Gcgr*^−/−^, and *Glp-1r/Gipr* double KO mice, compared with their WT controls. The triagonist-induced augmentation of GSIS was comparable between *Gipr*^−/−^ and WT mouse islets (Fig. 3a). A modest reduction in triagonist-induced insulin secretion was observed in *Gcgr*^−/−^ islets (Fig. 3b). Notably, a similar reduction in insulin secretion was observed in response to other secretagogues in *Gcgr*^−/−^ mouse islets. To rule out technical artefacts, we evaluated mono- and multi-agonist effects on GSIS in *Gcgr*^−/−^ and control mice, normalising insulin secretion to protein content. GCG-induced GSIS was significantly reduced in *Gcgr*^*−/−*^ mouse islets, whereas GLP-1-, GIP- and triagonist-induced insulin secretion remained comparable with insulin secretion in WT mice (Fig. 3c). Furthermore, the selective GCGR antagonist LY2409021 (1 µmol/l) effectively suppressed GCG-induced insulin secretion but had no impact on the triagonist-enhanced GSIS in WT islets (Fig. 3d). Surprisingly, the triagonist-induced augmentation of GSIS was completely abolished in *Glp-1r/Gipr* double KO islets (Fig. 3e). A similar response was obtained in WT islets treated with the GLP-1R antagonist exendin-3; at 1 µmol/l, exendin-3 suppressed triagonist-induced insulin secretion by up to 70% (Fig. 3f).

### Triagonist enhances GSIS primarily through the Gαq–TRPM5 signalling pathway

**Fig. 4:**
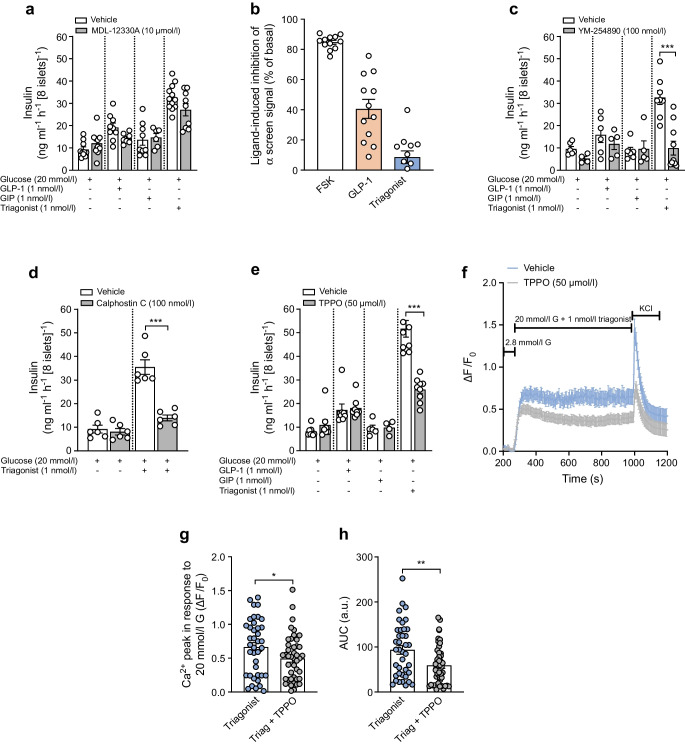
Triagonist improves GSIS in isolated islets through Gαq signalling and TRPM5 activation. (**a**) Insulin secretion (ng ml^−1^ h^−1^ [8 islets]^−1^) in isolated islets from WT mice on a chow diet (LFD) (*n*≥5 mice per condition, measured in duplicate). After 1 h pre-incubation in KRB with 2.8 mmol/l glucose in the presence or absence of 10 µmol/l MDL-12330A, islets were stimulated with 20 mmol/l glucose ± blocker, supplemented with mono- or multi-agonist (1 nmol/l each). Insulin content was determined by ELISA in supernatant fractions collected 60 min after stimulation. (**b**) Inhibition of cAMP-dependent α screen signals is shown after stimulation of islets with 10 µmol/l forskolin, 1 nmol/l GLP-1 or 1 nmol/l triagonist for 20 min at 37°C. Data from four independent experiments performed in triplicate are presented as mean ± SEM of ligand-induced inhibition of the α screen signal as % of basal. (**c**–**e**) Insulin secretion (ng ml^−1^ h^−1^ [8 islets]^−1^) in isolated islets from WT mice on LFD (*n*≥5 mice per condition, measured in duplicate) in the presence or absence of 100 nmol/l YM-254890, 100 nmol/l calphostin C or 50 µmol/l TPPO. (**f**) Intact islets from WT mice (*n*≥38 cells per condition, isolated from at least eight mice) were loaded with 3 µmol/l Fluo-4-AM and alterations in [Ca^2+^]_i_ of individual cells were monitored by confocal microscopy after increasing the extracellular glucose concentration from 2.8 to 20 mmol/l in the presence of triagonist (1 nmol/l) with (grey) or without (blue) 50 µmol/l TPPO. KCl (30 mmol/l) was used as a positive control. F_0_ was calculated as the mean fluorescence intensity during the final 2 min prior to stimulation with 20 mmol/l glucose + agonist. (**g**, **h**) Average of Ca^2+^ influx peaks assessed from baseline after glucose stimulation (**g**) and AUC (only during agonist application [**h**]). The data are presented as means ± SEM (circles in bar graphs represent single values) and statistical differences were assessed by unpaired two-tailed Student’s *t* test (**a**, **c**, **d**, **e**, **g**, **h**). *p<0.05, **p<0.01, ***p<0.001. a.u., arbitrary units; FSK, forskolin; G, glucose; Triag, triagonist

GLP-1R activation is known to engage both Gαs and Gαq signalling pathways. We next assessed their relative contributions towards triagonist-induced GSIS. The adenylate cyclase inhibitor MDL-12330A (10 μmol/l) caused only a modest reduction in GSIS responses to GLP-1 and triagonist in islets from WT mice fed an LFD (Fig. 4a). Next, we measured intracellular cAMP in WT mouse islets using an α screen assay to assess Gαs activation. The adenylate cyclase activator forskolin increased cAMP-dependent signals by over 80% relative to baseline (Fig. 4b). GLP-1 (1 nmol/l) raised cAMP levels by about 40%, whereas the triagonist did not significantly increase cAMP above baseline, suggesting that Gαs signalling may not be the primary pathway mediating triagonist-induced GSIS.

Next, we examined the role of Gαq signalling in triagonist-induced GSIS. Inhibition of Gαq with 100 nmol/l YM-254890 had no significant effect on GLP-1- or GIP-induced GSIS in islets from WT mice fed LFD (Fig. 4c). Notably, YM-254890 reduced triagonist-induced GSIS by up to 70% (Fig. 4c). It is worth noting that both GLP-1 and GIP elicited only modest insulin secretion, and the inhibitory effects of adenylate cyclase and Gαq blockers were not particularly pronounced. We believe these results are due to the nanomolar agonist concentrations applied, which were selected to simulate receptor activation levels similar to those that might occur physiologically, even if not precisely matching actual in vivo concentrations.

Given that PKC and TRPM5 are downstream effectors of Gαq signalling in beta cells, our next step involved determining whether the PKC inhibitor (calphostin C) and TRPM5 blocker (TPPO) suppress the triagonist-induced increase in GSIS. We demonstrated that 100 nmol/l calphostin C and 50 µmol/l TPPO each reduced the triagonist-induced GSIS increase by about 60% in primary islets (Fig. 4d, e). Next, we evaluated the impact of TPPO on triagonist-induced Ca^2+^ signalling in beta cells (Fig. 4f–h). TPPO significantly reduced the initial Ca^2+^ peaks induced by 20 mmol/l glucose in the presence of the triagonist and attenuated the sustained intracellular Ca^2+^ elevation in intact islets (Fig. 4g, h). Notably, the triagonist had no impact on [Ca^2+^] in the absence of extracellular Ca^2+^ (ESM Fig. 3). Nonetheless, Ca^2+^ release in response to the sarcoplasmic/endoplasmic reticulum (ER) calcium ATPase (SERCA) pump blocker cyclopiazonic acid demonstrated the sheer availability of ER calcium, ruling out the role of ER calcium in triagonist-induced Ca^2+^ signalling in beta cells.

### Dependence of triagonist-enhanced glycaemic effects on TRPM5 function

**Fig. 5:**
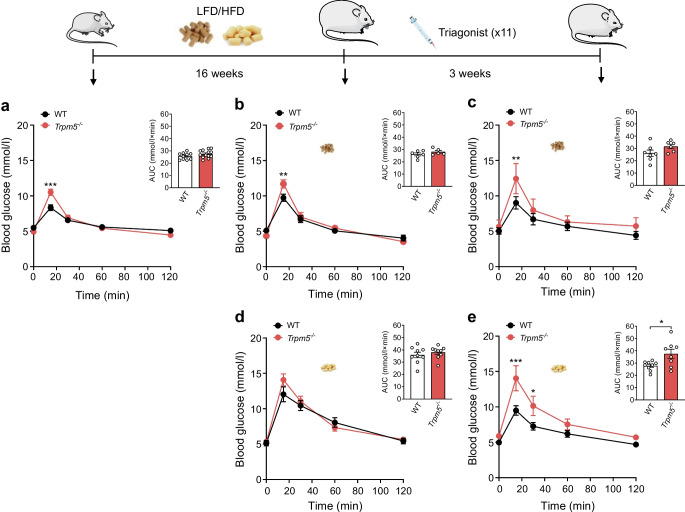
TRPM5 plays a crucial role in mediating the beneficial effects of triagonist on glucose metabolism in mice fed HFD. Ten- to twelve-week-old WT or *Trpm5*^−/−^ mice were divided into two groups: one maintained on a normal chow (LFD) (*n*≥6); and the other on D12451 (HFD) diet for 16 weeks (*n*≥9). Following 16 weeks of the respective diets, both groups received an i.p. injection of 3 nmol/kg triagonist every other day for 3 weeks (11 injections); GTT was performed before (**a**) and after the LFD (**b**) or HFD (**d**) and after the triagonist treatment in the LFD (**c**) and HFD (**e**) groups. For the GTT, mice were fasted overnight. Blood glucose levels before and within 2 h after i.p. injection of glucose (2 g/kg of body weight) in WT (black) and *Trpm5*^−/−^ mice (red) are shown, together with the corresponding AUC. Data show means ± SEM, and statistical differences were assessed by two-way ANOVA (blood glucose) or unpaired two-tailed Student’s *t* test (glucose AUC). Circles in bar graphs represent single values. *p<0.05, **p<0.01, ***p<0.001

**Fig. 6:**
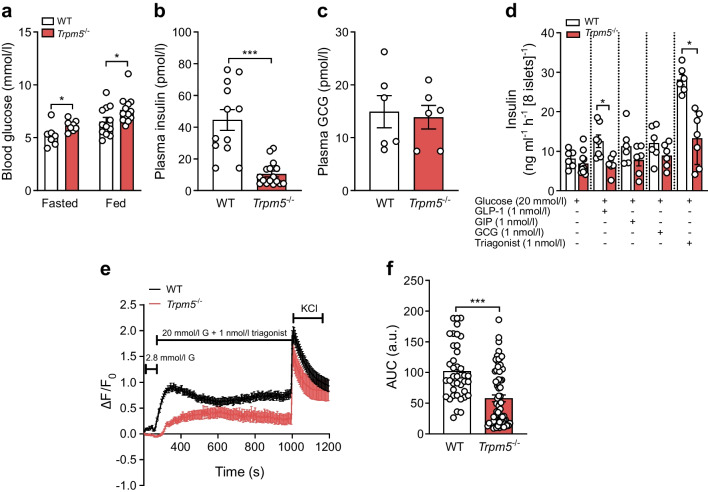
Triagonist improves glycaemic management in a TRPM5-dependent manner. (**a**) Blood glucose (mmol/l) in fasted (*n*≥7 mice per genotype) or freely fed (*n*≥12 mice per genotype) mice. (**b**, **c**) Plasma insulin levels (*n*≥12 mice per genotype, **b**) and plasma GCG levels (*n*≥6 mice per genotype, **c**) in freely fed male and female *Trpm5*^−/−^ mice and control littermates after 16 weeks of HFD and 3 weeks of treatment with triagonist. (**d**) Insulin secretion (ng ml^−1^ h^−1^ [8 islets]^−1^) was assessed in isolated islets of *Trpm5*^−/−^ and control littermate mice on HFD (*n*≥5 mice, measured in duplicate). After 1 h pre-incubation in KRB with 2.8 mmol/l glucose, islets were stimulated with 20 mmol/l glucose supplemented with mono- or multi-agonist (1 nmol/l each). Insulin content was determined by ELISA in supernatant fractions collected 60 min after stimulation. (**e**) Intact WT mouse islets (*n*≥40 cells per condition, isolated from at least four mice) and *Trpm5*^−/−^ islets (*n*≥30 cells per condition, isolated from at least four mice) after 16 weeks of HFD and 3 weeks of treatment with triagonist were loaded with 3 µmol/l Fluo-4-AM and alterations in [Ca^2+^]_i_ of individual cells were monitored by confocal microscopy after increasing the extracellular glucose concentration from 2.8 to 20 mmol/l in the presence of triagonist (1 nmol/l). KCl (30 mmol/l) was used as a positive control. F_0_ was calculated as the mean fluorescence intensity during the final 2 min prior to stimulation with 20 mmol/l glucose + agonist. (**f**) AUC was assessed only during agonist application. The data are presented as means ± SEM (circles in bar graphs represent single values) and statistical differences were assessed by unpaired two-tailed Student’s *t* test (**a**–**c**, **d**, **f**). *p<0.05, ***p<0.001. a.u., arbitrary units; G, glucose

To confirm that the therapeutic effects of the triagonist depend on TRPM5 function, we assessed whether its beneficial effects were diminished in *Trpm5*^−/−^ mice. The *Trpm5*^−/−^ mice exhibited slight glucose intolerance on LFD (Fig. 5a–c) and showed no significant changes in glucose tolerance after 16 weeks of HFD (Fig. 5d). The glucose-lowering effect of the triagonist observed in WT mice was significantly blunted in *Trpm5*^−/−^ mice after 16 weeks on HFD (Fig. 5e). Remarkably, triagonist-treated *Trpm5*^−/−^ mice showed significantly elevated fasting and fed blood glucose levels, accompanied by markedly reduced plasma insulin compared with their control littermates (Fig. 6a, b). The plasma levels of GCG remained comparable in both genotypes (Fig. 6c). Although the GSIS remained comparable in WT and *Trpm5*^−/−^ mouse islets, triagonist-induced GSIS was significantly reduced in *Trpm5*^−/−^ islets after 16 weeks on either LFD or HFD compared with controls (Fig. 6d and ESM Fig. 4). Importantly, triagonist-induced initial Ca^2+^ peaks and elevations in intracellular Ca^2+^ levels were markedly reduced in *Trpm5*^−/−^ mouse islets (Fig. 6e, f). Notably, only a slight impairment in glucose-induced Ca^2+^ responses was observed in *Trpm5*^−/−^ mouse islets (ESM Fig. 5a–c). The islet morphology, beta to alpha cell ratio, and the islet size remained comparable in the *Trpm5*^−/−^ mice and their control littermates (ESM Fig. 6a–c).

## Discussion

GLP-1, GIP and GCG are required to maintain normoglycaemic dynamics in healthy individuals. It is known that all three peptides enhance insulin secretion in WT mice, through mechanisms likely involving Gαs activation and cAMP production [19, 20]. However, a recent study demonstrated that in a mouse model of diabetes, GLP-1 exhibits preserved insulin secretion through a Gαq-dependent manner [13]. The shift from Gαs to Gαq effective coupling in persistently depolarised beta cells may hint at therapeutic and pathophysiological significance.

The triagonist IUB447 has shown superior efficacy compared with existing dual co-agonists and best-in-class mono-agonists in reducing body weight and improving glycaemic management in relevant rodent models. The enhanced insulin secretion induced by the triagonist is attributed to the simultaneous activation of all three receptors [1]. In the current study, we evaluated the significance of the GIPR and GCGR in triagonist-induced responses by employing *Gipr*^*−/−*^ and *Gcgr*^*−/−*^ mice. The results show that the triagonist functions independently of GIPR, as evidenced by fully intact triagonist enhancement of insulin secretion in *Gipr*^−/−^ mouse islets. The modest reduction in triagonist-induced insulin secretion observed in *Gcgr*^−/−^ mouse islets was mirrored by comparable reductions in insulin secretion observed with other secretagogues. Although we initially attributed this to impaired beta cell function, as reported in *Gcgr*^−/−^ mice [21], normalisation to protein content largely eliminated this effect. Additionally, pharmacological inhibition with LY2409021 indicated minimal involvement of GCGR in triagonist responses. Thus, we suggest that GIPR and GCGR are unlikely to be responsible for the enhanced effect of the triagonist on insulin secretion. These findings prompted us to hypothesise that GLP-1R may play a central role as the primary mediator of triagonist-induced responses in pancreatic islets. To test this, we employed a mouse model with a double *Glp-1r* and *Gipr* KO and a pharmacological approach using the specific GLP-1R antagonist exendin-3. Our data clearly demonstrate that the triagonist fails to enhance insulin secretion in the absence of functional GLP-1R activity. It is worth noting that the variability in agonist responses observed across different KO models may stem from differences in assay sensitivity, islet responsiveness and the physiological status of the mice, which can influence hormone responsiveness.

There is growing evidence indicating that GLP-1 activates both Gαq and Gαs signalling pathways, whereas the effects of GIP and GCG are mediated exclusively through Gαs [13]. Here, we report that the triagonist IUB447 did not enhance cAMP accumulation in pancreatic islets and blocking of the adenylate cyclase had a minimal impact on triagonist-induced insulin secretion. However, inhibition of Gαq signalling markedly suppressed the triagonist-induced enhancement of GSIS in WT mouse islets. A previous study showed that insulin secretion stimulated via the Gαq signalling pathway is enhanced in persistently depolarised beta cells. The Gαq agonist MK-2305 drastically improves glucose tolerance and insulin secretion in the KK-Ay mouse model of type 2 diabetes [13].

Our results attribute the triagonist-induced enhancement of GSIS to increased intracellular Ca^2+^ levels in beta cells. These elevations may also contribute to enhanced downstream signalling of Gαq through activation of phospholipase C (PLC), as has been reported in INS-1 cells [22]. This possibility is consistent with our previous observation in which the triagonist enhanced Ca^2+^ influx in the human pancreatic beta cell line 1.1B4, and inhibition of PLC activity with U73122 significantly attenuated the triagonist-induced increases in intracellular Ca^2+^ concentration [9]. Previous studies demonstrated that activation of PKC occurs downstream of Gαq-dependent activation of PLC in beta cells. It has been shown that the stimulatory effect of GLP-1 on insulin secretion is, to some extent, dependent on PKC, as evidenced by the ability of the PKC activator phorbol 12-myristate 13-acetate to mimic the effects of GLP-1 on electrical activity. Additionally, PKC inhibition completely abolishes the stimulatory effect of GLP-1 on insulin secretion [23]. Here, we show that PKC inhibition with calphostin C markedly reduces triagonist-induced GSIS enhancement in WT islets. Previous studies suggested that both PKC activation and increased intracellular Ca^2+^ levels are required for TRPM5 activation [24]. Thus, we explored the possible involvement of transient receptor potential channels in triagonist-induced insulin secretion. We previously reported that La^3+^ and Ruthenium Red, non-selective TRP channel blockers, strongly inhibited triagonist-enhanced GSIS and significantly suppressed triagonist-induced Ca^2+^ influx in 1.1B4 cells [9]. Here, we obtained responses similar to those caused by selective inhibition of TRPM5 with TPPO.

Modulation of TRPM5 activity plays a key role in regulating beta cell function and insulin secretion [25, 26]. *TRPM5* SNPs are often associated with disrupted insulin secretion, elevated plasma glucose levels and lower GLP-1 levels [27]. Previous studies suggested that TRPM5 activation and the subsequent increases in Na^+^-influx play a crucial role in GLP-1-induced increases in beta cell electrical activity [28]. Although GLP-1 increases the amplitude of the voltage-gated Ca^2+^ current, closure of K_ATP_ channels is not the sole mechanism by which GLP-1 stimulates beta cell electrical activity. In both mouse and human beta cells, the ability of GLP-1 to induce membrane depolarisation and initiate action potential firing depend on extracellular Na^+^ and TRPM5 activity [23, 29, 30]. Importantly, the GLP-1-activated Na^+^ current cannot initiate electrical activity and insulin secretion unless the K_ATP_ channels are almost fully inhibited [23]. This is crucial for ensuring the safety of GLP-1-based drug therapy, as the stimulatory effect of such agents is glucose-dependent, and clarifies the rationale behind the minimal impact of the triagonist, even at significantly elevated concentrations, on insulin secretion under condition of low glucose.

In the current study, we showed that *Trpm5*^−/−^ mice fed HFD display glucose intolerance and impaired insulin response to GLP-1. This finding is consistent with a previous study in which mice lacking TRPM5 function exhibited a decline in glucose tolerance and an impaired GSIS [31, 32]. Given our ex vivo findings that triagonist-induced insulin secretion is impaired by TRPM5 inhibition, we examined the effects of triagonist on glycaemic management in vivo in the absence of this channel. Here, we showed that triagonist treatment failed to reverse the impaired glucose tolerance in *Trpm5*^−/−^ mice. Furthermore, fasted and fed blood glucose levels were considerably higher in *Trpm5*^−/−^ mice than in their WT counterparts after triagonist treatment. This observation suggests the importance of TRPM5 function in mediating triagonist-induced increases in insulin secretion and overall normoglycaemia.

In this study, we report that the insulinotropic effect of the triagonist IUB447 is mainly due to its direct binding to the GLP-1R, leading to an increase in both Gαq signalling and TRPM5 activity. This observation agrees with our previously reported homology model, which indicated that the triagonist binds to the GLP-1R in the same binding pocket, in a very similar fashion to its native peptide agonist, GLP-1 [9]. A closer look into this model reveals several putative hydrogen bonds and a salt bridge that the triagonist forms in addition to those that are already present when GLP-1 binds in that pocket (see PDB ID 5VAI [33]). These additional bonds likely result in a stronger and longer binding of the triagonist to the GLP-1R when compared with GLP-1. These putative additional triagonist-specific bonds, beyond those that are identical to those formed when GLP-1 binds, include a possible salt bridge formation between Glu139 in the receptor and Arg23 of the triagonist (Gln23 in GLP-1) as well as hydrogen bonds between Arg190 in the receptor and Gln9 of the triagonist (Glu9 in GLP-1) and also between Arg121 in the receptor and Asp34 of the triagonist (Lys34 in GLP-1). Such a feature could affect the spatial adjustments of other elements during GLP-1R activation, potentially leading to improved therapeutic outcomes, as suggested by others [34]. Although our findings indicate that GLP-1R is the primary mediator of the insulinotropic effect of the triagonist in murine islets, receptor engagement may differ in human islets. Given that species-specific differences have been reported for other multi-agonists such as tirzepatide [35], further studies using human islets are warranted to confirm the involvement of GLP-1R and the Gαq–TRPM5 signalling axis in humans. Furthermore, it is important to note that our findings here specifically reflect the pharmacological profile of the triagonist IUB447, characterised by Finan et al. [1], and should not be extrapolated to structurally distinct triagonists such as SAR441255 [36] or LY3437943 [37], which may differ in receptor bias, potency and in vivo efficacy.

## Supplementary Information

Below is the link to the electronic supplementary material.

ESM (PDF 901 KB)

## Data Availability

All data will be shared by the corresponding author upon reasonable request.

## Abbreviations

[Ca^2+^]_i_: Intracellular Ca^2+^ concentration
ER: Endoplasmic reticulum
GCG: Glucagon
GCGR: Glucagon receptor
GIP: Glucose-dependent insulinotropic polypeptide
GIPR: Glucose-dependent insulinotropic polypeptide receptor
GLP-1: Glucagon-like peptide-1
GLP-1R: Glucagon-like peptide-1 receptor
GSIS: Glucose-stimulated insulin secretion
HFD: High-fat diet
LFD: Normal chow diet
KO: Knockout
nt: Nucleotide
PKC: Protein kinase C
PLC: Phospholipase C
TPPO: Triphenylphosphine oxide
TRPM5: Transient receptor potential melastatin 5
WT: Wild-type
