# Supplementary material for: GLP-1/GIP/GCG receptor triagonist (IUB447) enhances insulin secretion via GLP-1 receptor and Gαq signalling pathway in mice
Source: Diabetologia. 2025 Sep 9;68(11):2567–80. doi: 10.1007/s00125-025-06525-0 (PMC12534261; doi:10.1007/s00125-025-06525-0)
Supplement: Supplementary file 1 — ESM (PDF 901 KB) [file 125_2025_6525_MOESM1_ESM.pdf]

Electronic Supplementary Material

ESM Tables

ESM Table 1 Source information for used chemicals and reagents

Chemicals and Reagents	Manufacturer
AlphaScreen cAMP Detection Kit	Revvity, Waltham, MA, USA
Bovine Serum Albumin (BSA)	Carl Roth, Karlsruhe, Germany
$CaCl_2$	Sigma Aldrich, St. Louis, MO, USA
Calphostin C	Sigma Aldrich, St. Louis, MO, USA
Collagenase-P	Roche, Mannheim, Germany
D12331	Research Diets, New Brunswick, NJ, USA
D12451	Research Diets, New Brunswick, NJ, USA
D-Glucose	Sigma Aldrich, St. Louis, MO, USA
DMEM	Gibco, Grand Island, NY, USA
DPBS	Gibco, Grand Island, NY, USA
Eosin	Sigma Aldrich, St. Louis, MO, USA
Exendin-3	Sigma Aldrich, St. Louis, MO, USA
FBS	Gibco, Grand Island, NY, USA
FBS Superior	Sigma Aldrich, St. Louis, MO, USA
Fluo-4-AM	Invitrogen, Waltham, MA, USA
Forskolin	Sigma Aldrich, St. Louis, MO, USA
GIP	Sigma Aldrich, St. Louis, MO, USA
GLP-1	Sigma Aldrich, St. Louis, MO, USA
Glucagon	Sigma Aldrich, St. Louis, MO, USA
Glucagon ELISA assay	Mercodia, Uppsala, Sweden
Haematoxylin	Sigma Aldrich, St. Louis, MO, USA
HBSS	PAN-Biotech, Aidenbach, Germany
HEPES	Sigma Aldrich, St. Louis, MO, USA
HEPES Buffer Solution	Gibco, Grand Island, NY, USA
IBMX	Sigma Aldrich, St. Louis, MO, USA
Insulin ELISA assay	ALPCO, Salem, NH, US
KCl	Sigma Aldrich, St. Louis, MO, USA
KH_2PO_4	Sigma Aldrich, St. Louis, MO, USA
Lithium heparin LH micro sample tubes	Sarstedt, Nümbrecht, Germany
LY2409021	MedChemExpress, Monmouth Junction, NJ, USA
MDL-12330A	Sigma Aldrich, St. Louis, MO, USA
MEGAclear Kit	Thermo Fisher Scientific, Waltham, MA, USA
$MgSO_4$	Sigma Aldrich, St. Louis, MO, USA
NaCl	Sigma Aldrich, St. Louis, MO, USA
$NaHCO_3$	Sigma Aldrich, St. Louis, MO, USA
NLS-Cas9 protein	PNA Bio Inc., Newbury Park, CA, USA
One Step Mouse Genotyping Kit	Vazyme, Nanjing, China
Penicillin (100 U/ml) and streptomycin (100 μg/ml)	Gibco, Grand Island, NY, USA
RPMI 1640	Thermo Fisher Scientific, Waltham, MA, USA
Sodium Pyruvate	Gibco, Grand Island, NY, USA
TPPO	Sigma Aldrich, St. Louis, MO, USA
Triagonist	DOI: 10.1038/nm.3761
YM-254890	MedChemExpress, Monmouth Junction, NJ, USA
β-Mercaptoethanol	Gibco, Grand Island, NY, USA

ESM Table 2 sgRNAs for generating KO mice

**Target**	**sgRNA sequence**
Gipr gRNA1	GGCTTTGTCTTCCGCCAGTG
Gipr gRNA2	GGTCTCTCCAAGATCCCCAC
Gipr gRNA3	CTGCAGATCATGTATACCGT
Gipr gRNA4	CCTGCAGATCATGTATACCG
Glp-1r gRNA1	TAGACTCTTCACACTCCGAC
Glp-1r gRNA2	CTGTGCAGAACCGGTACACA
Glp-1r gRNA3	CCACTGTGTAGATAATGTAC
Glp-1r gRNA4	GATGGCTGAAGCGATGACCA
Gcgr gRNA1	CCAGCAGGAGTACTTGTCGA
Gcgr gRNA2	GTGGTACCAAGGTAGGTACC
Gcgr gRNA3	CATTGGGAGGCGTTGCGCCA
Gcgr gRNA4	TCAAGAGGTGTGGGCCCGAT

ESM Table 3 Primers for mice genotyping

Primer	Sequence
Glp-1r F	TGCGATTCCTGTTACTAACTCA
Glp-1r R	AGTGAGAAGGACCCTCTGGTT
Gcgr F	GTGCCTTGGGCAAACACAAA
Gcgr R	AGGAGCCACACCAATGTACC
Gipr F	AGCTGATCTCGGGTGAGGATs
Gipr R	TGTGGCGATCAGAGGTCAAC
Gipr -9nt F	TCGTCAGGGACAGGGAGTAG
Gipr -9nt R	CAGTGATGGAGTGATCTTGGAG
Gipr WT R	CAAGATCCCCACTGGCCATC
Trpm5 WT F	CTAGACACACGGTAGACAGAGTCAG
Trpm5 WT R	CCTGTCGGATTTCCTCCAGCACCAG
Trpm5 mut F	GACGAGTTCTTCTGAGGGGATCGATC

F and R indicate forward and reverse primers, respectively.

ESM Table 4 Genotyping PCR protocol for *Trpm5*

Temperature [°C]	Time	Cycles
95	5 min	
95	30 s	10
70	30 s	
72	1 min	
95	30 sec	35
59	30 sec	
72	1 min	
72	5 min	
8	∞	

ESM Table 5 KRB-Buffer recipe

Chemical	Concentration
NaCl	115 mmol/l
KCl	4.5 mmol/l
KH_2PO_4	1.2 mmol/l
$CaCl_2$	2.6 mmol/l
$MgSO_4$	1.2 mmol/l
HEPES	10 mmol/l
$NaHCO_3$	20 mmol/l
BSA	0.1% (w/v)

Set pH to 7.4

ESM Table 6 Dilution and source information for used antibodies

**Antigen**	**Host species**	**Dilution**	**Source**	**Catalogue no.**
Glucagon	Mouse	1:1000	Sigma Aldrich, St. Louis, MO, USA	G2654
Insulin	Guinea pig	Ready to use	Agilent, Santa Clara, CA, USA	IR002
Alexa Fluor 594 goat anti-guinea pig	Goat	1:1000	Thermo Fisher Scientific, Waltham, MA, USA	A11076
Alexa Fluor 488 goat anti-mouse	Goat	1:1000	Thermo Fisher Scientific, Waltham, MA, USA	A11001

ESM Figures

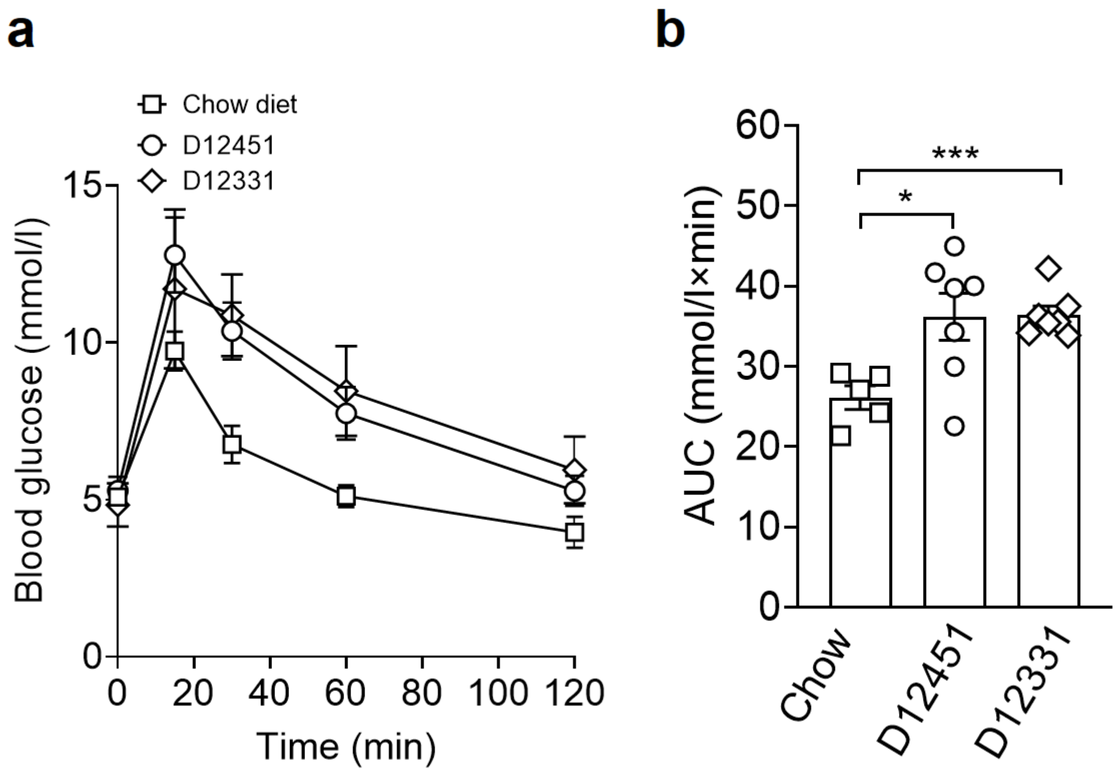

ESM Fig. 1 WT mice subjected to a 16 week (2 different) HFD (D12451, D12331) exhibit marked glucose intolerance. **a** and **b** compare the traces presented in Figure 1a–c. Ten- to twelve-week-old C57BL/6 mice were divided into three groups: One group maintained on a normal chow diet (LFD) (*n*=7), while the other two groups were placed on different HFD — either D12451 or D12331 — for 16 weeks (*n*=7 per group). For the GTT, mice were fasted overnight. (**a**) Blood glucose levels (mmol/l) before and within 2 h after i.p. injection of glucose (2 g/kg of body weight). (**b**) The area under the curves (AUC in mmol/l × min). Data show means ± SEM, and statistical differences were assessed by two-way ANOVA (a) or one-way ANOVA (b). *$p < 0.05$, ***$p < 0.001$

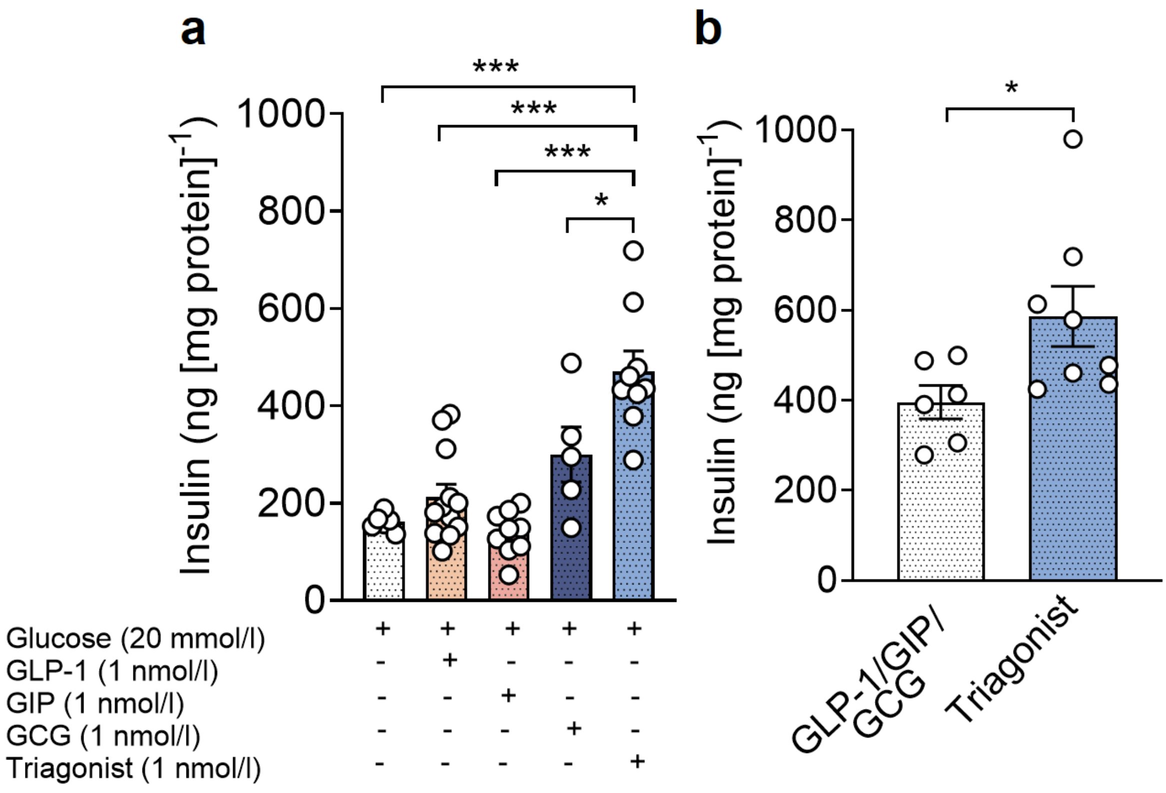

ESM Fig. 2 Triagonist enhances GSIS in the pancreatic beta cell line (MIN6 cells). Insulin secretion (ng [mg protein]$^{-1}$) was determined in MIN6 cells (5 independent experiments performed in duplicate). After 1 h pre-incubation in KRB with 2.8 mmol/l glucose, MIN6 cells were stimulated in 20 mmol/l glucose supplemented with mono- or multi-agonist (1 nmol/l each). Insulin content in supernatants were collected 60 min after stimulation. ELISA was used to determine the insulin content in the supernatant. Data show means ± S.E.M., and statistical differences were assessed by one-way ANOVA (a) and unpaired two-tailed Student’s *t* test (b). Circles in bar graphs represent single values. $*p < 0.05$, $***p < 0.001$

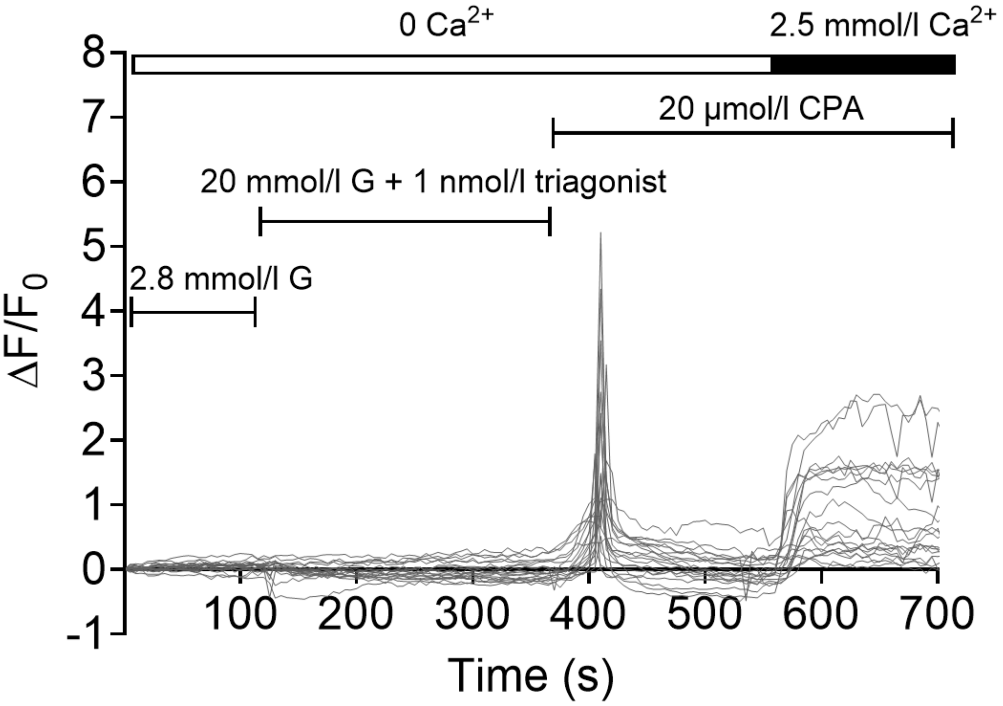

ESM Fig. 3 In the absence of extracellular Ca^{2+}, triagonist application has no effect on cytosolic Ca^{2+}. Individual traces of Fluo-4 fluorescence intensity in single islet cells in the presence of triagonist, recorded by excitation at λ 480 nm. The addition of 1 nmol/l triagonist together with 20 mmol/l glucose did not affect cytosolic Ca^{2+} concentration in the absence of extracellular Ca^{2+}. EGTA was applied as a Ca^{2+} chelator. $[Ca^{2+}]_i$ responses were monitored following store depletion with 20 μmol/l CPA in the absence of extracellular Ca^{2+} and subsequent re-addition of 2.5 mmol/l Ca^{2+} (SOCE). CPA, cyclopiazonic acid; G, glucose

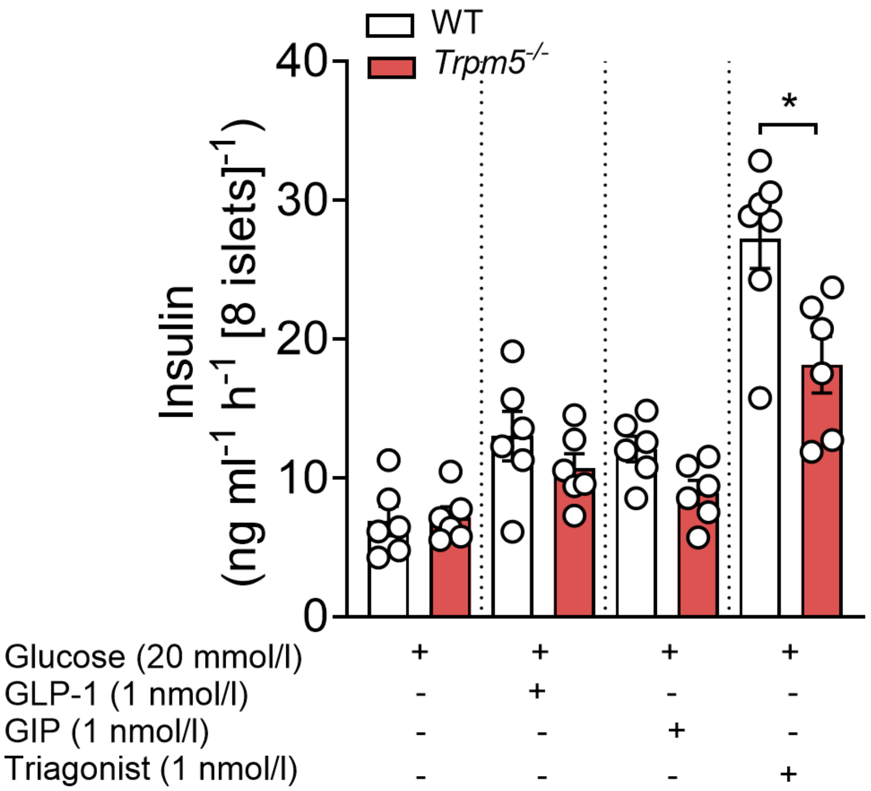

ESM Fig. 4 Triagonist improves GSIS in a TRPM5-dependent manner. Insulin secretion (ng ml^{-1} h^{-1} [8 $islets]^{-1}$) was assessed in isolated islets of *Trpm5*$^{-/-}$ and control littermate mice on a chow diet (LFD) (n=3 mice, measured in duplicate). After 1 h pre-incubation in KRB with 2.8 mmol/l glucose, islets were stimulated in 20 mmol/l glucose supplemented with mono- or multi-agonist (1 nmol/l each). Insulin content in supernatants were collected 60 min after stimulation and ELISA was used to determine the insulin content in the supernatant. The data are presented as means ± SEM (circles in bar graph represent single values) and statistical differences were assessed by unpaired two-tailed Student's t test. $*p < 0.05$

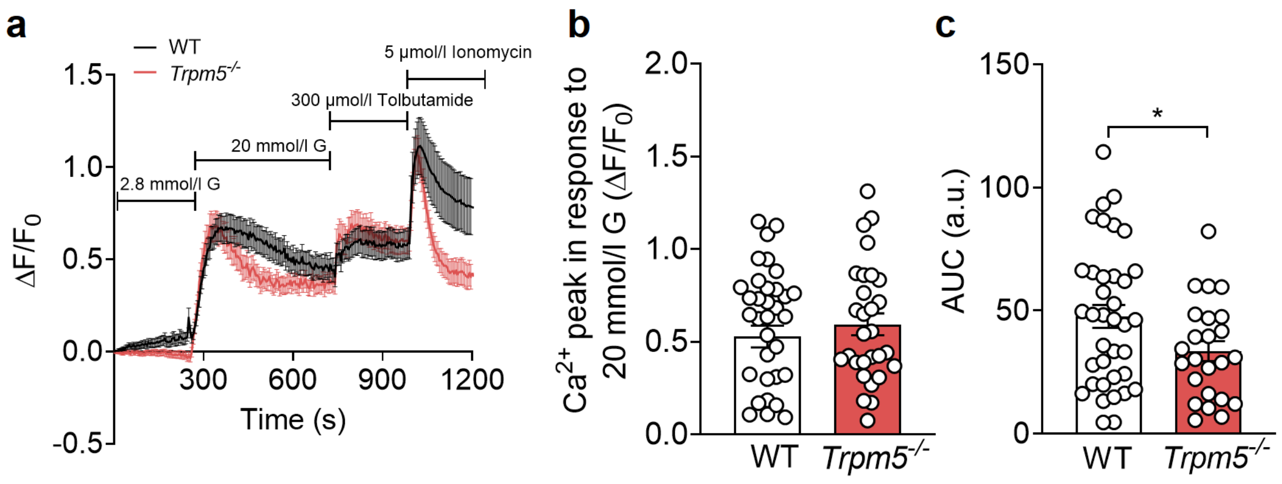

ESM Fig. 5 The increase in glucose-induced Ca^{2+} levels is marginally impaired in isolated islets of *Trpm5*$^{-/-}$ mice. (**a**) Intact islets isolated from WT and *Trpm5*$^{-/-}$ mice (n≥40 cells per condition, 5 mice per genotype) were loaded with 3 µmol/l Fluo-4-AM and alterations in $[Ca^{2+}]_i$ of individual cells were monitored by confocal microscopy after increasing the extracellular glucose concentration from 2.8 to 20 mmol/l and applying 300 µmol/l tolbutamide. Ionomycin (5 µmol/l) was used as a positive control. (**b** and **c**) Average of Ca^{2+} influx peaks assessed from baseline after glucose stimulation and area under the curve (only during 20 mmol/l glucose application). The data are presented as means ± SEM (circles in bar graphs represent single values) and statistical differences were assessed by unpaired two-tailed Student's *t* test (b, c). $*p < 0.05$. a.u., arbitrary units; G, glucose

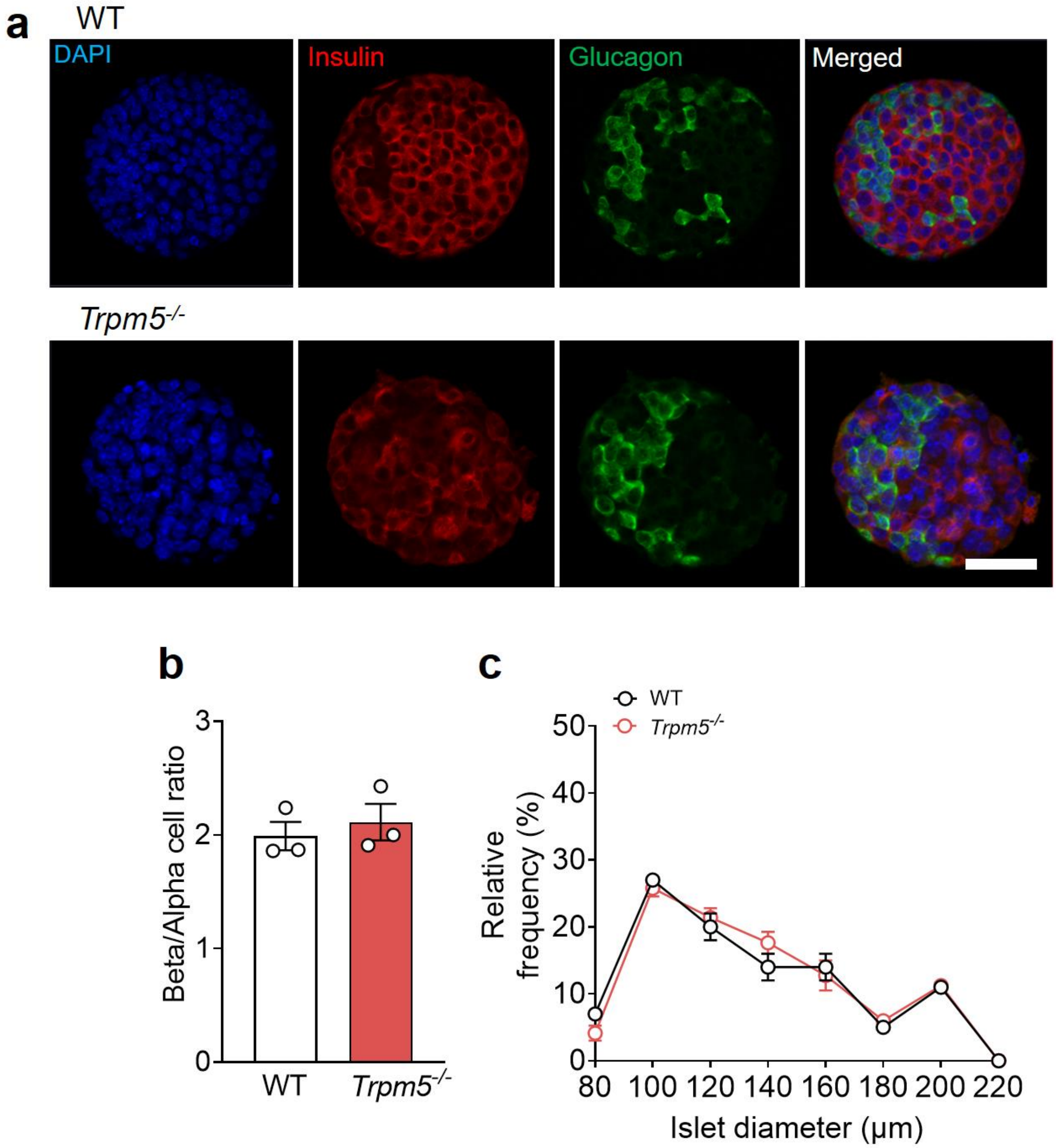

ESM Fig. 6 Morphology of WT and *Trpm5*$^{-/-}$ pancreatic islets. (**a**) Immunofluorescent insulin (red) and glucagon (green) staining of pancreatic cryosections of WT and *Trpm5*$^{-/-}$ mice. Nuclei were stained with DAPI (blue) and scale bars represent 100 µm. (**b**) Quantification of the ratio of the number of beta and alpha cells per pancreatic islet in WT and *Trpm5*$^{-/-}$ mice (3 mice per genotype). (**c**) Relative frequency plot of islet diameter comparing WT with *Trpm5*$^{-/-}$ islets (3 mice per genotype). Data are given as means ± SEM (circles in bar graph represent single values), and statistical differences were assessed by unpaired two-tailed Student's *t* test (b).
